# Antitumor activity of photodynamic therapy performed with nanospheres containing zinc-phthalocyanine

**DOI:** 10.1186/1477-3155-11-41

**Published:** 2013-12-16

**Authors:** Flávia Arruda Portilho, Cláudio Eduardo de Oliveira Cavalcanti, Ana Luisa Miranda-Vilela, Luciana Landim Carneiro Estevanato, João Paulo Figueiró Longo, Maria de Fátima Menezes Almeida Santos, Anamélia Lorenzetti Bocca, Olímpia Paschoal Martins, Andreza R Simioni, Paulo César Morais, Ricardo Bentes Azevedo, Antonio Claudio Tedesco, Zulmira Guerrero Marques Lacava

**Affiliations:** 1 Instituto de Ciências Biológicas, Departamento de Genética e Morfologia, Universidade de Brasília, CEP: 70910-970 Brasília, DF, Brazil; 2 Faculdade de Medicina (FAMED), Universidade Federal de Alagoas, Maceió, AL 57072-970, Brazil; 3 Universidade Estadual de Ciências da Saúde de Alagoas (UNCISAL), Maceió, AL 57010-300, Brazil; 4 Faculdades Integradas da União Educacional do Planalto Central (Faciplac), Campus Gama, Curso de Medicina, Brasília, DF, Brazil; 5 Instituto de Ciências Biológicas, Departamento de Biologia Celular, Universidade de Brasília, Brasília, DF 70910-900, Brazil; 6 Departamento de Química, Laboratório de Fotobiologia e Fotomedicina, Centro de Nanotecnologia e Engenharia Tecidual, Faculdade de Filosofia, Ciências e Letras de Ribeirão Preto, Universidade de São Paulo, Ribeirão Preto, SP 14040-901, Brazil; 7 Instituto de Física, Núcleo de Física Aplicada, Universidade de Brasília, Brasília, DF 70910-900, Brazil; 8 School of Automation, Huazhong University of Science and Technology, Wuhan 430074, China

**Keywords:** Photodynamic therapy, Ehrlich tumor, Drug delivery system, Albumin nanospheres, Zinc-phthalocyanine, Doxorubicin

## Abstract

**Background:**

The increasing incidence of cancer and the search for more effective therapies with minimal collateral effects have prompted studies to find alternative new treatments. Among these, photodynamic therapy (PDT) has been proposed as a very promising new modality in cancer treatment with the lowest rates of side effects, revealing itself to be particularly successful when the photosensitizer is associated with nanoscaled carriers. This study aimed to design and develop a new formulation based on albumin nanospheres containing zinc-phthalocyanine tetrasulfonate (ZnPcS_4_-AN) for use in the PDT protocol and to investigate its antitumor activity in Swiss albino mice using the Ehrlich solid tumor as an experimental model for breast cancer.

**Methods:**

Ehrlich tumor’s volume, histopathology and morphometry were used to assess the efficacy of intratumoral injection of ZnPcS_4_-AN in containing tumor aggressiveness and promoting its regression, while the toxicity of possible treatments was assessed by animal weight, morphological analysis of the liver and kidneys, hemogram, and serum levels of total bilirubin, direct bilirubin, indirect bilirubin, aspartate aminotransferase (AST), alanine aminotransferase (ALT), gamma glutamyl transferase (GGT), alkaline phosphatase, creatinine and urea. In order to evaluate the efficacy of PDT, groups of animals treated with intratumoral injection of doxorubicin (Dox) were also investigated.

**Results:**

Intratumoral injection of ZnPcS_4_-AN was found to be efficient in mediating PDT to refrain tumor aggressiveness and to induce its regression. Although tumor volume reduction was not significant, PDT induced a remarkable increase in the necrosis area seen in the tumor’s central region, as in other experimental groups, including tumor and Dox treated groups, but also in the tumor’s peripheral region. Further, PDT showed minimal adverse effects. Indeed, the use of ZnPcS_4_-AN in mediating PDT revealed anti-neoplastic activity similar to that obtained while using intratumoral Dox therapy.

**Conclusions:**

PDT mediated by the new formulation ZnPcS_4_-AN enhanced the inhibition of tumor growth while producing practically no adverse effects and thus emerges as a very promising nanotechnology-based strategy for solid cancer treatment.

## Background

The increasing incidence of cancer and the search for the development of more effective therapies with minimal side effects have prompted studies to find alternative new treatments. Among new therapies, photodynamic therapy (PDT) appears as a promising modality in cancer treatment with the lowest rates of side effects. Since the 1990s PDT has been investigated in basic research and showed to be more effective when nanostructured materials are used as the drug delivery system (DDS) for the active compounds [1-7]. Nowadays, interest in using PDT is increasing as a result of its recognition by the FDA (U.S. Food and Drug Administration) and other world agencies as an effective therapy for the treatment of several diseases, including cancer [8]. PDT is currently being used in the clinical phase for treatment of several types of tumors [9,10] around the world [11-14]. However, this therapy is particularly effective in the treatment of easily accessible lesions, such as non-melanoma skin cancers and lesions with well-defined borders [2], although it has been used outside oncology applications, including ophthalmology, dermatology, cardiology, virus inactivation, and blood purification [1].

PDT consists in the systemic or topical application of a photosensitizer that, after a specific time interval for distribution, displays preferential accumulation in the neoplastic tissue. Subsequently, photosensitizer molecules are irradiated with laser light of a particular wavelength to induce photochemical and photophysical phenomena, marked by the occurrence of energy transfer to the nearby oxygen, generating reactive oxygen species as singlet oxygen, hydroxyl radical, and superoxide anions. As a result of PDT-induced oxidative stress, cellular organelles and membranes become damaged, a process recognized as tumor photodamage. Singlet oxygen causes microvascular acute injury and blood vessel blockage in tumor and induces apoptosis of tumor cells, achieving the purpose of local treatment [4,15-17].

Successful pre-clinical and clinical studies resulted in the first officially approved photosensitizing drug for use in PDT of selected tumors, known as Photofrin^®^, a semi-purified hematoporphyrin derivative [1-3]. Following Photofrin^®^ other first-generation photosensitizers have also been approved [1,2]. In addition, some second-generation photosensitizers are now being submitted to clinical testing in research centers in various countries, including Brazil [5,14,17]. The second generation compounds have been designed to improve the uptake of selective tumor cells while taking advantage of the increased depth penetration of light with a longer wavelength than that used to activate Photofrin^®^. Second-generation photosensitizers are generally pure, can be activated by light in the wavelength range of 630–800 nm, and all share a lower incidence of prolonged cutaneous photosensitivity compared to Photofrin^®^[1]. Among them, phthalocyanines absorb light between 630 and 700 nm and provide maximum tissue penetration, thus presenting properties for tumor localization and high efficiency as a photosensitizing agent [18,19]. The most favorable photophysical properties of phthalocyanines to be used in PDT are dependent upon the core metal ion which comprises their molecular structure. They can be attached to metals such as zinc or aluminum, forming zinc-phthalocyanine or chloride-aluminum-phthalocyanine complexes [20,21].

Most photosensitizers which are used clinically or in preclinical development are hydrophobic and strongly aggregate in aqueous media, including phthalocyanines. Aggregation significantly reduces the photosensitizing efficacy as only monomeric species are appreciably photoactive [15,21]. The aggregation tendency may be minimized by using a nanostructured photosensitive molecular structure and also through the non-covalent binding of photosensitizers to a carrier molecule, such as albumin [21]. While photosensitizers can reach the lesion more easily, and thus selectively accumulate in tumor tissues, molecular association with albumin may be performed in physiological solutions, thus conferring biocompatible characteristics to the formulation [20]. To further enhance the selective release and accumulation of photosensitizers at the targeted tissue, various carriers and drug delivery systems have been investigated [1,5,14,21-25].

Aiming to potentialize the outcomes while using nanostructured drug delivery systems carrying phthalocyanines as photosensitizers, in the present study we report on the fabrication of a new albumin-based nanosphere containing zinc-phthalocyanine tetrasulfonate (ZnPcS_4_-AN) and further investigate its antitumor activity while mediating photodynamic therapy. To conveniently evaluate the new ZnPcS_4_-AN for PDT efficacy, groups of animals treated with intratumoral injection of doxorubicin (Dox) were also investigated.

## Results

### ZnPcS_4_-AN characterization

**Figure 1:**
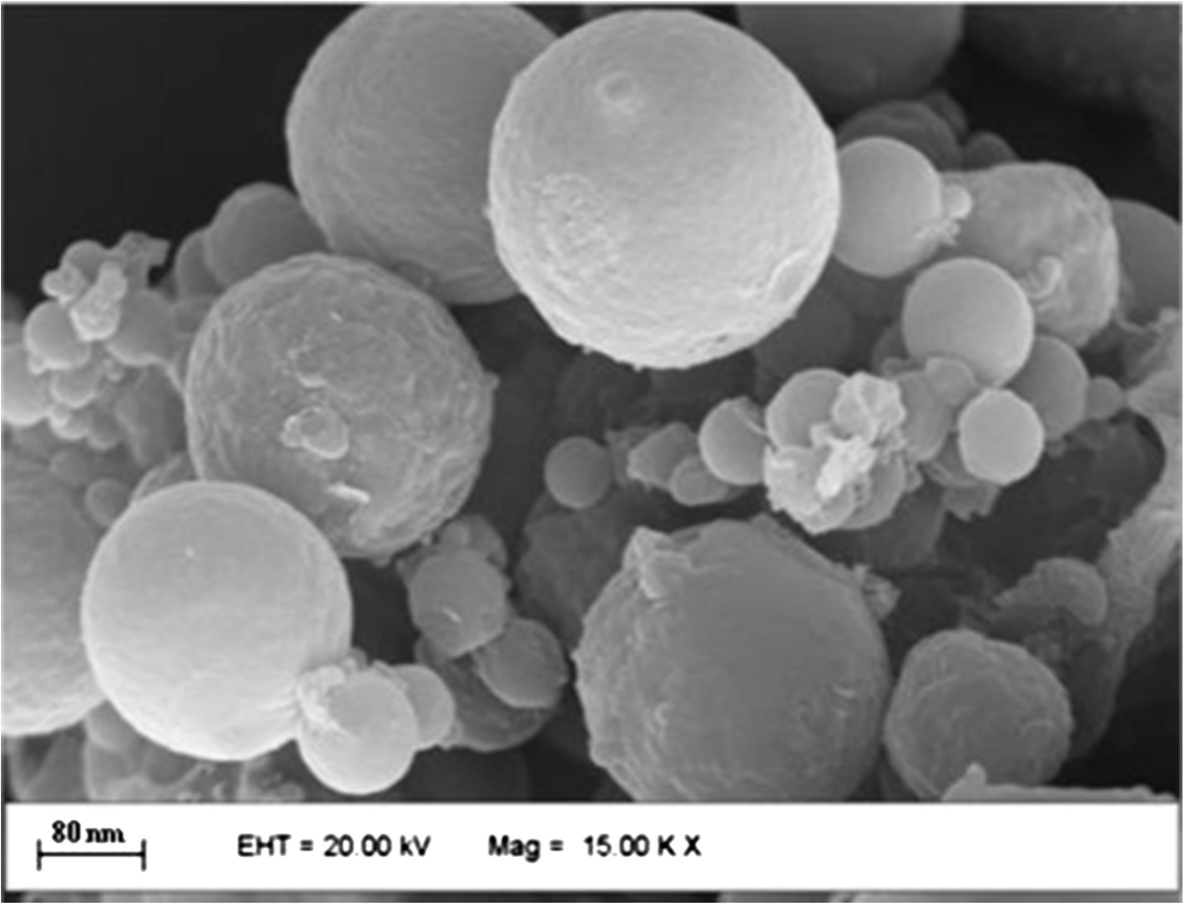
**Scanning electron micrograph of sample ZnPcS**_**4**_**-AN.** The photomicrography shows the spherical shape of the nanospheres with smooth surface.

Scanning electron micrographs of the ZnPcS_4_-AN sample reveal the spherical shape (Figure 1) with smooth surface, as expected for this kind of system [26,27], whereas the analysis of the nanosphere size reveals that the employed preparation method produced nanospheres with average diameter of 320 nm.

**Figure 2:**
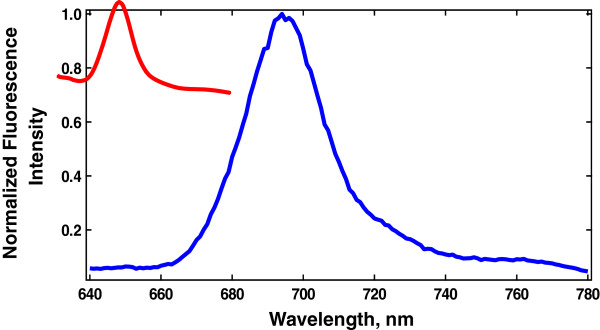
**Normalized fluorescence emission spectrum of ZnPcS**_**4 **_**(-) and ZnPcS**_**4**_**-AN (-) in PBS.** ZnPcS_4-_AN presents a small blue shift of the peak emission in comparison to the characteristic peak emission of ZnPcS_4_at 690 nm.

Fluorescence spectra were obtained by exciting the samples (suspended in phosphate buffer) at 612 nm while the emission was recorded in the range of 640–780 nm (Figure 2). Bandwidths were fixed at 5 nm for excitation and emission.

As observed in Figure 2 encapsulation of the ZnPcS_4_ within the BSA-based nanosphere leads to a small blue shift of the peak emission in comparison to the characteristic peak emission of the free photosensitizer at 690 nm, which corresponds to the maximum fluorescence emission wavelength of ZnPcS_4_.

The obtained results indicate that the drug delivery system did not affect the photophysical and phobiological properties of the ZnPcS_4_ in the ground and excitated states, responsible for the complex photophysical pathway that leads to the biological response by PDT treatment [26,27]. In other words, the ZnPcS_4_ maintains its activity with the advantage of being encapsulated in a biocompatible system, in this case the BSA-based nanospheres. The final analysis developed in the ZnPcS_4_-AN sample are based on zeta potential and particle size analyses.

The zeta potential data and the hydrodynamic particle size evaluation support the observed physico-chemical stability and reveal the size homogeneity of the as-prepared submicron-sized polymeric particles (see Table 1). No appreciable variation on the average values of zeta potential and hydrodynamic particle size characteristics were observed up to 30 days of samples evaluations. As shown in Table 1 the ZnPcS_4_-AN sample presented negative zeta potential values. This picture is consistent with the high colloidal stability of ZnPcS_4_-AN sample upon dispersion and the high negative value of the zeta potential [28].

**Table 1:** **Characterization of ZnPcS**_**4**_**-AN sample**

**Sample**	**Hydrodynamic diameter (nm)**	**Size dispersion (μm)**	**Zeta potential (mV)**
ZnPcS_4_-AN	238.0 ± 0.3	11.00 ± 0.04	-38.6 ± 0.4

### Tumor dimensions, histology, and percentage of necrosis

**Figure 3:**
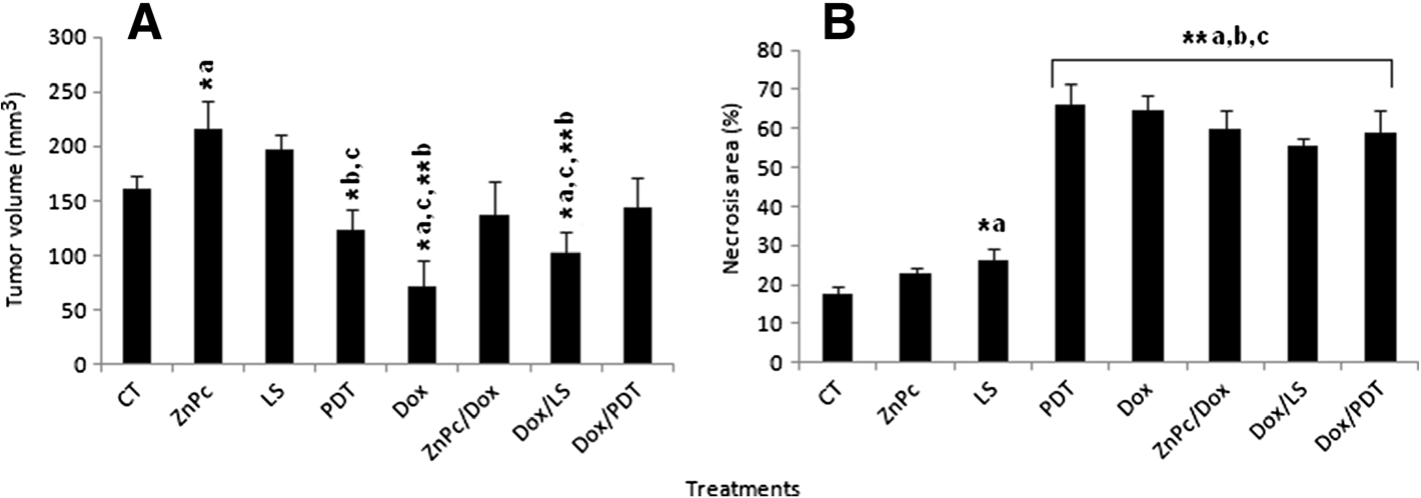
**Tumor characteristics after photodynamic therapy and doxorubicin treatment.** Sections refer to: **(A)** tumor volume and **(B)** percentage of necrosis. Bar graphs were expressed as SEM (standard error of mean). Asterisks indicate significant (*p < 0.05) and highly significant (**p < 0.01) differences detected by the Mann Whitney U test, with a = significant compared to CT group; b = significant compared to ZnPc group; c = significant compared to LS group. For groups’ nomenclature see Table 5.

Photodynamic therapy performed with the albumin-based nanospheres containing zinc-phthalocyanine tetrasulfonate (ZnPcS_4_-AN) (PDT group) showed no significant tumor-volume reduction (Figure 3A) when compared to non-treated tumor (CT group), whereas treatments with doxorubicin (Dox group) and doxorubicin associated with laser exposure (Dox/LS group) caused a significant reduction in tumor-volume. However, morphometric evaluation showed the highest level of necrosis after photodynamic therapy using ZnPcS_4_-AN (PDT group, 66 ± 5% necrosis). Significant necrosis was also observed with laser treatment (LS group, 26 ± 3% necrosis), and all doxorubicin groups: Dox (65 ± 4% necrosis), ZnPc/Dox (60 ± 4% necrosis), Dox/LS (56 ± 2% necrosis) and Dox/PDT (59 ± 6% necrosis) (Figure 3B).

**Figure 4:**
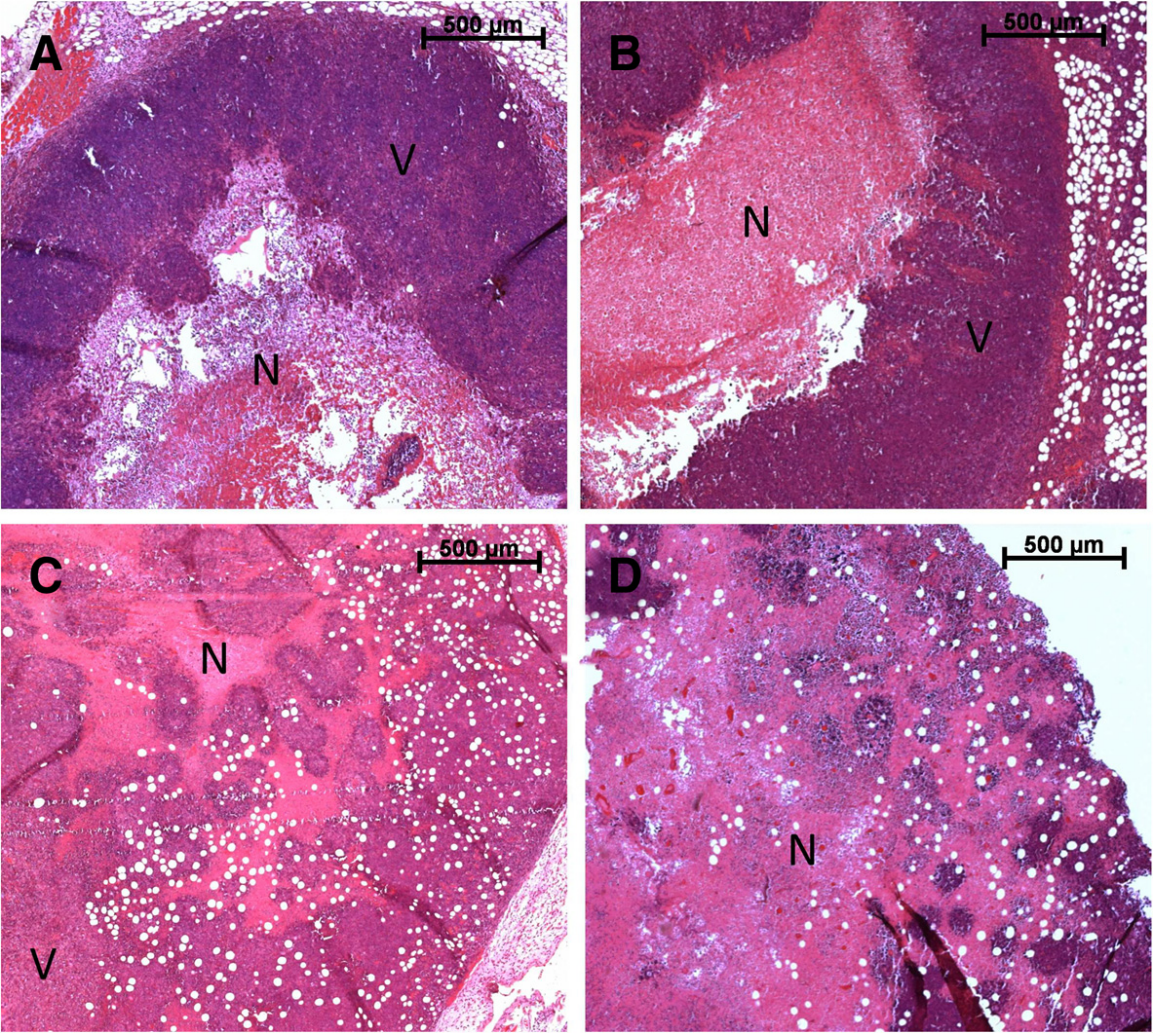
**Ehrlich tumor histopathology after photodynamic therapy and doxorubicin treatment.** Sections refer to: **(A)** control tumor, **(B)** albumin-ZnPc treated tumors, and **(C)** doxorubicin treated tumor presenting central area of tumor necrosis (N) and viable cells in the tumor periphery (V). Section **(D)** represents the tumor treated with PDT, showing the necrotic tissue in the central and peripheral areas of the tumor.

Histological sections from control Ehrlich tumor (CT group) showed a typical pattern consisting of a peripheral area of viable tumor layers surrounding a central area of necrotic tissue. Viable tumor tissues showed the presence of intact nucleus, cytoplasm limits and isolated mitotic figures, as identified by the hematoxylin staining. In contrast, necrotic tissues were characterized by an amorphous eosinophilic mass without cytoplasmic membrane integrity and absence of cell nucleus. Necrotic areas were also highly infiltrated by defense cells, especially neutrophils (data not showed), which were also identified in all experimental groups, especially in the central areas. Differences in variations of necrotic area distribution inside tumors were identified among the PDT group and other experimental groups, including the control tumors (CT, ZnPc, and LS) and the tumor groups treated with doxorubicin (Dox, ZnPc/Dox, Dox/LS, and Dox/PDT). We found that photodynamic therapy induced the presence of necrotic tissue in both central and peripheral areas of tumor sections (Figure 4).

### Histology of liver and kidneys

**Figure 5:**
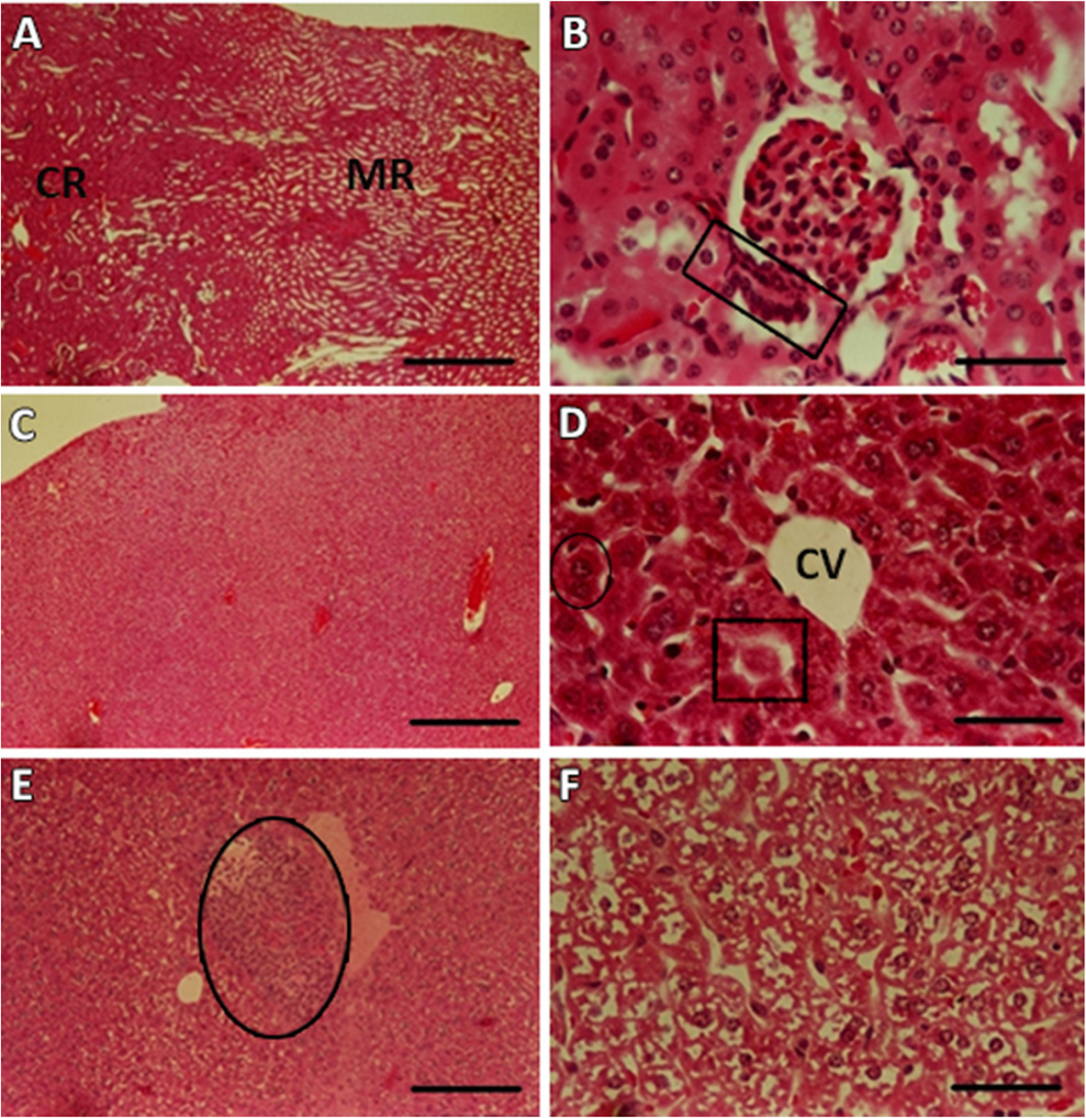
**Kidney and liver photomicrographs after photodynamic therapy and doxorubicin treatment. (A)**, **(B)**, **(C)** and **(D)**: Negative control (NC); **(E)** and **(F)**: Doxorubicin treated group (**Dox**). Sections: **(A)** Cortical (CR) and medular (MR) regions of the kidney. Bar = 250 μm; **(B)** renal corpuscle in the center. In the detail (rectangle), the macula densa. Bar = 50 μm. **(C)** General aspect of hepatic tissue. Bar = 250 μm. **(D)** CV: centrolobular vein, binucleate hepatocyte (circle) and sinusoids (square). **(E)** Inflammatory infiltrate next to the vein observed in a Dox treated animal. **(F)** Degenerative process of the liver. For **(D)**, **(E)** and **(F)**, Bar = 50 μm.

Macroscopically, all animals (including the PDT group) presented kidneys with normal appearance characterized by pink-red coloration and firm consistency. By light microscopy, only two animals treated with Dox and subjected to PDT (Dox/PDT group) presented some organ alteration, showing mild mononuclear inflammatory infiltration with predominantly focal distribution (data not showed). Animals from all groups also showed liver of normal appearance in macroscopic terms, with reddish brown coloration and firm consistency. By light microscopy, only the liver of the Dox group presented some alteration, with few inflammatory cells inside the vessel near the vascular wall and hepatocytes with cytoplasmic vacuolation (Figure 5).

### Hematology

#### ***Erythrogram***

A significant reduction in red blood cells (RBC) and hematocrit (HCT) was observed after the PDT and also in almost all treatments compared to the negative control, except in the Dox and Dox/PDT groups for RBC and Dox group for HCT. However, except for the ZnPc group, RBC values remained inside the reference values for mice (7.3-10.5 × 10^6^/μL) [29]. It is noteworthy that this was the only change in erythrogram parameters induced by PDT, although the components of PDT (ZnPc and LS) may cause some other alterations. The ZnPc group, as well as the Dox/LS group, also presented significantly decreased hemoglobin (HGB) values. Mean corpuscular hemoglobin (MCH) fell significantly for the CT, LS and ZnPc groups, while the mean corpuscular hemoglobin concentration (MCHC) fell in CT, LS, Dox, ZnPc/Dox, and Dox/PDT treatment groups. No significant differences with respect to the negative control were observed for mean corpuscular volume (MCV) or red cell distribution width (RDW) (Table 2).

**Table 2:** **Effects of ZnPcS**_**4**_**-AN-based PDT and/or Dox treatments on erythrogram parameters of healthy and Ehrlich solid tumor-bearing mice**

**Group**	**Treatment**	**RBC (x 10**^**6**^**/μL)**	**HGB (g/dL)**	**HCT (%)**	**MCV (fL)**	**MCH (pg)**	**MCHC (g/dL)**	**RDW (%)**
**1**	**NC**	8.8 ± 0.1	12.8 ± 0.1	31.5 ± 0.3	35.8 ± 0.1	14.5 ± 0.1	40.5 ± 0.2	13.01 ± 0.09
**2**	**CT**	8.6 ± 0.2	12.0 ± 0.2	30.7 ± 0.5	35.7 ± 0.3	13.9 ± 0.1**^a^	38.9 ± 0.2**^a^	13.6 ± 0.3
**3**	**ZnPc**	6.6 ± 0.7**^a.^*^b^	9.2 ± 0.9**^a.^*^b^	23.3 ± 2.5**^a. ^*^b^	35.6 ± 0.4	14.2 ± 0.3	39.9 ± 0.5	14.1 ± 0.7
**4**	**LS**	8.5 ± 0.1*^a.c^	11.9 ± 0.2**^c^	30.5 ± 0.5*^a.^**^c^	36.0 ± 0.1	14.08 ± 0.08**^a^	39.1 ± 0.2**^a^	13.1 ± 0.2
**5**	**PDT**	8.1 ± 0.2**^a.^*^b.d^	11.4 ± 0.3**^c^	29.0 ± 0.6**^a.^*^b^	35.7 ± 0.2	14.0 ± 0.1	39.2 ± 0.2	14.0 ± 0.3
**6**	**Dox**	7.8 ± 0.3	11.1 ± 0.3	30 ± 1	38 ± 2	14.1 ± 0.3	38 ± 1**^a^	13.5 ± 0.4
**7**	**ZnPc/Dox**	8.0 ± 0.4*^a^	10.9 ± 0.5	28 ± 1**^a^	35.25 ± 0.05	13.60 ± 0.00*^a.d^	38.50 ± 0.00*^a.c.d^	13.8 ± 0.4
**8**	**Dox/LS**	7.6 ± 0.4**^a.^*^b.d^	10.9 ± 0.5*^a^	27 ± 1*^a.d.^**^b^	35.8 ± 0.7	14.3 ± 0.1*^b^	39.9 ± 0.5	13.5 ± 0.6
**9**	**Dox/PDT**	8.3 ± 0.2^.^*^c.h^	11.8 ± 0.2**^c^	30.1 ± 0.6*^a.^**^c^	36.2 ± 0.3	14.18 ± 0.12*^g^	39.2 ± 0.1**^a.^*^g^	14.2 ± 0.3
	**P-values**	0.000	0.000	0.000	0.195	0.018	0.000	0.033

Data were expressed as mean ± SEM (standard error of mean). RBC = Red Blood Cells; HGB = Hemoglobin; HCT = Hematocrit; MCV = Mean Corpuscular Volume; MCH = Mean Corpuscular Hemoglobin; MCHC = Mean Corpuscular Hemoglobin Concentration; RDW = Red cell Distribution Width (represents an indication of the amount of variation - anisocytosis - in cell size); g/dL = grams per deciliter; fL = fentoliters; pg = picograms. P-values of HGB, MCV and RDW were generated by ANOVA, while other p-values were generated by Kruskal-Wallis test. Lower-case letters indicate significant diferences in 2-by-2 comparisons detected by Bonferroni (HGB, MCV and RDW) or Mann–Whitney (other variables) tests, with a = significant compared to group 1; b = significant compared to group 2; c = significant compared to group 3; d = significant compared to group 4; e = significant compared to group 5; f = significant compared to group 6; g = significant compared to group 7; h = significant compared to group 8. Asterisks indicate significant differences (*p < 0.05) or highly significant (**p < 0.01). For details of experimental groups see Table 5.

### Leukogram

**Figure 6:**
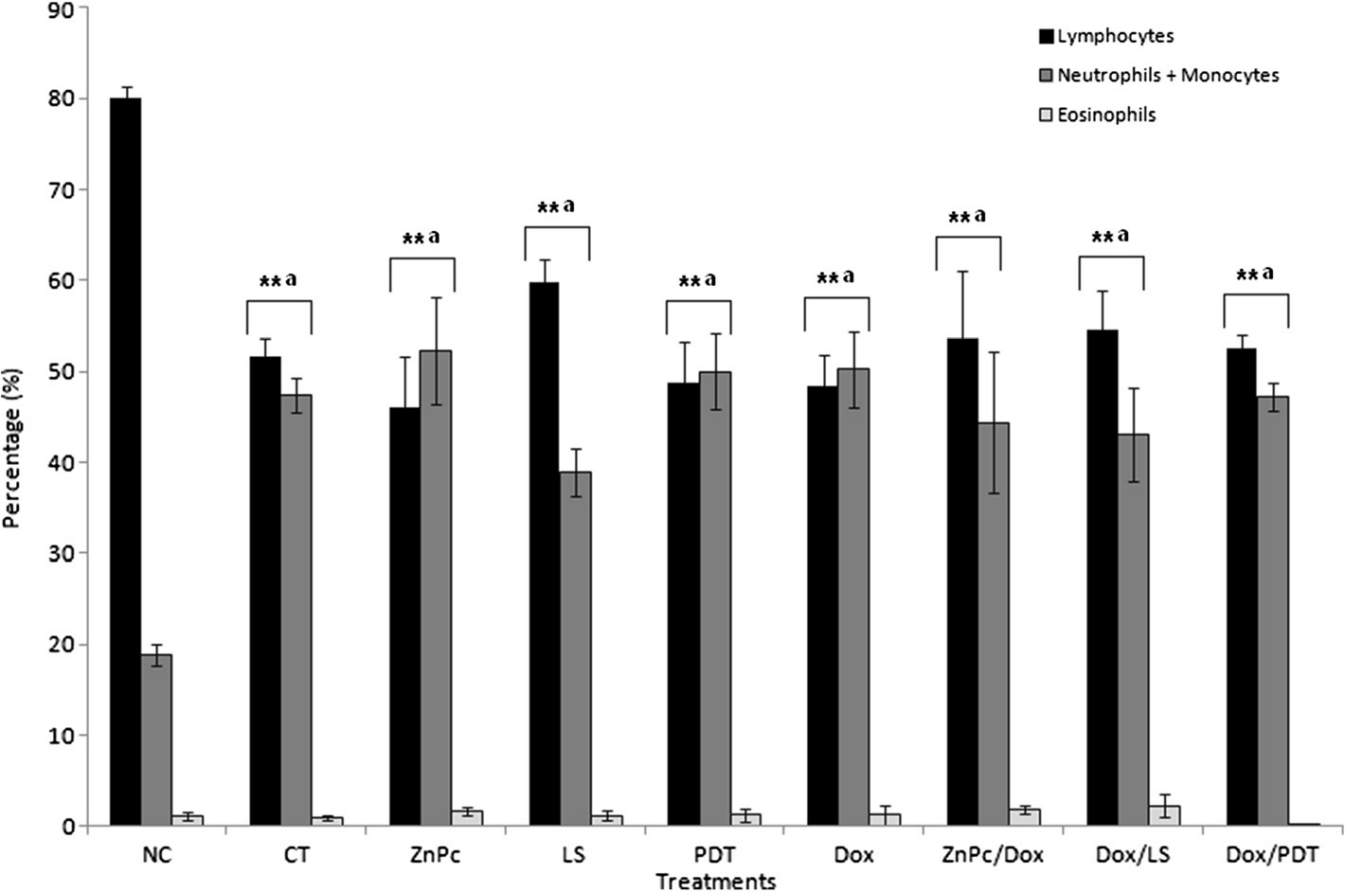
**Percentage of differential leukocyte counts after PDT and DOX treatments.** Negative control (NC) received filtered water and no tumor implantation took place. In the control tumor (CT), tumor was implanted and no treatment was performed. Bar graphs were expressed as SEM (standard error of mean). Asterisks indicate highly significant differences (**p < 0.01) detected by the Bonferroni test, with a = significant compared to NC group. For groups’ nomenclature see Table 5.

Control tumor and all treatment groups, including the PDT group, presented a significant decrease in lymphocytes with a concomitant increase in neutrophils + monocytes compared to healthy animals (negative control), whereas no significant change was observed for eosinophils (Figure 6).

### Plateletgram

With respect to the negative control and except for Dox and Dox/PDT, significantly increased platelets were observed for control tumor, PDT group and almost all other treatment groups. The PDT group also showed another alteration: a significant increase in the platelet large cell ratio (P-LCR), also observed for ZnPc and LS. Alterations in other plateletgram parameters were not observed after PDT. Mean platelet volume (MPV) significantly increased only for the ZnPc group, whereas platelet distribution width (PDW) increased for ZnPc, Dox/LS, and Dox/PDT groups. However, no conclusive result was obtained for the ZnPc/Dox group, as the plateletgram could only be evaluated in one animal (Table 3).

**Table 3:** **Effects of ZnPcS**_**4**_**-AN-based PDT and/or Dox treatments on plateletgram parameters of healthy and Ehrlich solid tumor-bearing mice**

**G**	**Treatment**	**PLT (× 10**^**3**^**/μL)**	**MPV (fl)**	**P-LCR (%)**	**PDW (fl)**
**1**	**NC**	1172 ± 50	6.31 ± 0.06	6.4 ± 0.3	6.77 ± 0.04
**2**	**CT**	1496 ± 50**^a^	6.5 ± 0.1	8.1 ± 0.9	6.80 ± 0.07
**3**	**ZnPc**	1503 ± 300*^a^	7.0 ± 0.1**^a^	9.4 ± 0.5**^a^	7.3 ± 0.2*^a^
**4**	**LS**	1366 ± 70*^a^	6.54 ± 0.07	7.8 ± 0.5*^a^	6.86 ± 0.07
**5**	**PDT**	1528 ± 100**^a^	6.7 ± 0.1	8.4 ± 0.6*^a^	6.9 ± 0.1
**6**	**Dox**	1312 ± 200	6.6 ± 0.3	8 ± 1	6.9 ± 0.3
**7**	**ZnPc/Dox**^**(1)**^	1219	7.10	12.70	6.90
**8**	**Dox/LS**	1808 ± 90**^a.^*^b.d^	6.3 ± 0.2*^c^	7 ± 2	6.53 ± 0.03**^a.^*^b.c.d^
**9**	**Dox/PDT**	1390 ± 80*^h^	6.3 ± 0.1**^c^	6 ± 1**^c^	6.6 ± 0.1*^a.c^
	**P-values**	0.005	0.000	0.026	0.035

Data were expressed as mean ± SEM (standard error of mean). G = Group; PLT = Platelet Count; MPV = Mean Platelet Volume; P-LCR = Platelet Large Cell Ratio; PDW = Platelet Distribution Width; fl = fentoliters. P-values of MPV were generated by ANOVA, while other p-values were generated by Kruskal-Wallis test. Lower-case letters indicate significant diferences in 2-by-2 comparations detected by Bonferroni (MPV) or Mann–Whitney test (other variables), with a = significant compared to group 1; b = significant compared to group 2; c = significant compared to group 3; d = significant compared to group 4; h= significant compared to group 8. Asterisks indicate significant differences (*p < 0.05) or highly significant (**p < 0.01).

^(1)^Corresponds to the count of only one animal (the other samples coagulated). It was not used in statistical analyses of plateletgram. For details of experimental groups see Table 5.

### Biochemical analyses

Although significant differences (with respect to the negative control) were observed for the direct and indirect bilirubin, aspartate aminotransferase (AST), alkaline phosphatase, creatinine and urea (Table 4) in several treatment groups, including the non-treated tumor control (CT group), photodynamic therapy (PDT group) caused significant alterations only in the indirect bilirubin and alkaline phosphatase evaluations. It is noteworthy that although the PDT components (ZnPc and LS groups) presented significant alterations for direct bilirubin and urea when compared to the negative control group (NC), similar alterations were not observed in the PDT group. The biochemical values observed after doxorubicin treatment (Dox group) were not significantly different from the PDT group data. However, when PDT and Dox are combined (Dox/PDT group), direct bilirubin is higher than in the PDT group, although not differing from the NC group.

**Table 4:** **Effects of ZnPcS**_**4**_**-AN-based PDT and/or Dox treatments on biochemical parameters of healthy and Ehrlich solid tumor-bearing mice**

**G**	**T**	**Total bilirubin (mg/dL)**	**Direct bilirubin (mg/dL)**	**Indirect bilirubin (mg/dL)**	**AST (U/L)**	**ALT (U/L)**	**GGT (U/L)**	**Alkaline phosphatase (U/L)**	**Creatinine (mg/dL)**	**Urea (mg/dL)**
**1**	**NC**	0.10 ± 0.00	0.04 ± 0.00	0.06 ± 0.00	71 ± 4	35 ± 2	1.88 ± 0.09	115 ± 9	0.20 ± 0.03	59 ± 2
**2**	**CT**	0.10 ± 0.00	0.03 ± 0.00**^a^	0.08 ± 0.00**^a^	186 ± 30**^a^	36 ± 3	1.8 ± 0.3	68 ± 8**^a^	0.20 ± 0.08	57 ± 5
**3**	**ZnPc**	0.10 ± 0.00	0.02 ± 0.00**^a^	0.08 ± 0.00**^a^	236 ± 30**^a^	44 ± 3	2.00 ± 0.00	41 ± 6**^a. ^*^b^	0.16 ± 0.02	46 ± 3**^a^
**4**	**LS**	0.10 ± 0.00	0.02 ± 0.00**^a^	0.08 ± 0.00**^a^	96 ± 20*^c^	33 ± 6	2.3 ± 0.3	42 ± 9**^a. ^*^b^	0.44 ± 0.09*^a.b.c^	39 ± 9*^a^
**5**	**PDT**	0.10 ± 0.00	0.02 ± 0.00	0.08 ± 0.00**^a^	162 ± 80	30 ± 9	1.9 ± 0.1	44 ± 7**^a^	0.29 ± 0.05	44 ± 8
**6**	**Dox**	0.10 ± 0.00	0.03 ± 0.00*^d^	0.07 ± 0.00*^d^	174 ± 20**^a^	37 ± 4	1.5 ± 0.3*^c.d^	53 ± 10	0.25 ± 0.06	55 ± 3
**7**	**ZnPc/Dox**	0.10 ± 0.00	0.02 ± 0.00**^a^	0.08 ± 0.00**^a^	160 ± 10**^a^	27 ± 2	1.8 ± 0.3	47 ± 10**^a^	0.25 ± 0.03*^c^	50 ± 3
**8**	**Dox/LS**	0.10 ± 0.00	0.03 ± 0.00*^d^	0.07 ± 0.00*^d^	162 ± 20**^a.^*^d^	35 ± 4	2.00 ± 0.00	88 ± 20*^c.d^	0.18 ± 0.03	56 ± 5
**9**	**Dox/PDT**	0.3 ± 0.2	0.04 ± 0.01*^d.e^	0.2 ± 0.2	106 ± 20*^a.b.f.^**^c^	25 ± 4	3 ± 1	42 ± 6	0.20 ± 0.02	39 ± 6
	**P-values**	0.393	0.000	0.001	0.000	0.247	0.519	0.000	0.067	0.025

Data were expressed as mean ± SEM (standard error of mean). G = Group; T = Treatment; AST = Aspartate Aminotransferase; ALT = Alanine Aminotransferase; GGT = Gamma-Glutamyl Transferase; mg/dL = miligrams per deciliters; ULl = Units per liter. P-values of ALT were generated by ANOVA, while other values were generated by Kruskal-Wallis test. Lower-case letters indicate significant diferences in 2-by-2 comparations detected by Bonferroni (ALT) or Mann–Whitney (other variables) tests, with a = significant compared to group 1; b = significant compared to group 2; c = significant compared to group 3; d = significant compared to group 4; e = significant compared to group 5; f = significant compared to group 6. Asterisks indicate significant differences (*p < 0.05) or highly significant (**p < 0.01 or p < 0.001). For details of experimental groups see Table 5.

## Discussion

Cancer research has been conducted in order to find an appropriate treatment for each type of neoplasm. PDT is considered an effective method for early treatment of cancer and a palliative treatment of advanced cancer [30]. Using a combination of nontoxic photosensitizing chemical and visible light to destroy the tumor through the generation of reactive oxygen species [30], PDT presents advantages over chemotherapy, such as lower toxicity and higher safety in the treatment of malignant lesions [31]. However, there are limitations on the use of PDT, as the efficacy of the treatment depends on the light penetration into the target tissue and also on the photophysical and photochemical characteristics of the chosen photosensitizing agent [32].

Surface decoration of nanoparticles represents a crucial factor in biomedical applications, such as when colloidal stability is needed for longer periods of time, or for preventing particle agglomeration and premature precipitation. The pharmacokinetic characteristics of zinc phthalocyanine (ZnPc), such as long residence in the activated state and intense absorption in the visible red band, make this molecule a very promising second-generation photosensitizer for use in PDT [33]. However, to avoid its agglomeration in aqueous media, particularly the tendency to aggregate in physiological solution, the use of a non-covalent binding of ZnPc to a carrier template is highly recommended [1,20-22]. Since cancer cells overexpress the albumin receptor to favor the capture of the nutrient albumin [21], the use of albumin polymers as carriers of ZnPc may offer the advantage of increasing absorption of the photosensitizer by tumor cells, in addition to preventing degradation of photosensitizer before it achieves its biological effect [23]. Our findings demonstrated that albumin nanospheres containing zinc-phthalocyanine tetrasulfonate were indeed efficient in mediating PDT for tumor remission, in very good agreement with the literature [34]. Ehrlich tumor is a very aggressive tumor that spontaneously presents a central necrosis process [35,36]. Due to its aggressive pattern, this tumor type can be maintained in ascitic forms as a source of tumor cells for subsequent subcutaneous tissue injections to produce solid tumors. Although histological sections of Erhlich tumor in the present study had a typical pattern that consisted of a peripheral area of viable tumor layers surrounding a central area of necrotic tissue [37], necrosis areas were found in both the central area and in the peripheral regions of the tumor in the group treated with PDT, where the presence of inflammatory infiltrates and hemorrhages in blood vessels was also observed. Similar results were also reported by Longo et al. [25]. Since there was a significant increase in the percentage of necrotic area in the PDT group, the observed infiltration of inflammatory cells after PDT procedure was possibly responsible for the slight reduction in tumor volume when compared to the control tumor. Moreover, tumor volume of animals treated with PDT was not statistically different from treatment with Dox alone. These results, combined with tumor morphometry findings, suggest that the efficacy of PDT in reducing the tumor was similar to the outcomes obtained while using Dox therapy.

Doxorubicin is a cytotoxic anthracycline antibiotic widely used in clinical practice for treatment of hematological malignancies and solid tumors, including breast cancer [38-40]. The anticancer effects of doxorubicin are mainly due to its ability to intercalate within DNA, to inhibit topoisomerase II and to modify helicases dissociating duplex DNA into single stranded DNA, thus preventing DNA replication. Further, it also generates reactive oxygen species (ROS) that can damage macromolecules and lipid membranes [41,42]. Although the vast body of clinical experience considers systemic chemotherapy with doxorubicin to be a well tolerated and effective choice for most potentially anthracycline-sensitive tumors [43], its cardiotoxicity combined with the risk of promoting hepatic dysfunction [44] can limit its clinical application. Thus, although the use of anthracyclines is standard in antitumor activities [38,39], in an attempt to reduce adverse and increase therapeutic effects intratumoral injection of doxorubicin has been used both in patients [45,46] and rats [47] with malignant brain tumors, as well as in rat breast tumor models [48]. As a consequence, intratumoral injection of doxorubicin was also employed in this study to make the most appropriate comparison with the protocol for the use of the new ZnPcS_4_-AN photosensitizer complex for PDT.

Considering that (1) the mononuclear inflammatory infiltration had a predominant focal distribution only in kidneys of two animals treated with Dox and PDT combined therapies; (2) the cytoplasmic vacuolation of hepatocytes was observed only in the group treated with Dox; and (3) PDT was as effective in controlling tumor growth as Dox, it may be stated that ZnPcS_4_-AN mediated-PDT can enhance the inhibition of tumor growth while producing reduced side effects and thus representing a very promising strategy for tumor treatment. Due to the fact that tumor microvasculature has a discontinuous and loose nature, favoring its spread throughout the blood stream [49], serum and blood cell assays can be used to evaluate the effects of the employed treatments.

Biochemical tests are widely used for diagnosis of animal diseases [50,51], as the analyzed enzymes are generally intracellular and tissue-specific, and their profile fluctuation in the bloodstream may reflect cellular injury, increased membrane permeability or cell proliferation [51]. In combination with hemogram, which can be used to identify anemia, inflammatory, allergic and blood clotting disorders [52,53] biochemical tests represent important tools for pathologic analysis.

Alanine aminotransferase (ALT) is found in higher concentrations only in the liver. Thus, increased serum levels of this particular enzyme are used to evaluate hepatic lesion [54]. However, in the present study none of the treatments employed promoted significant differences in serum ALT compared to healthy animals. This indicates that while the cytoplasmic vacuolation of hepatocytes observed in animals treated with intratumoral injection of Dox could represent cytoplasmic degeneration, this was probably a mild and circumscribed event, corroborating the previous suggestion that intratumoral injection of Dox can reduce its adverse effects while increasing its therapeutic effects [45,46].

Elevated levels of indirect bilirubin are usually observed in situations of increased red blood cell lysis, whereas elevated levels of direct bilirubin are associated with intrahepatic cholestasis or extrahepatic bile duct obstruction [53]. In this context, the significant increase in indirect bilirubin with respect to the negative control observed for the tumor control and most of the treatments could be suggestive of hemolysis. Results of erythrogram, platelets and AST corroborate this assumption, since there is a significant reduction of red blood cells and a significant increase in platelets and in serum AST observed for the same groups, compared to healthy animals (negative control). However, except for the ZnPc group (one of the PDT controls), which showed HGB values below the reference values, all other groups presented values of the erythrogram within the reference values, despite the significant differences observed. Thus, results suggest that the tumor implantation was responsible for most of the observed changes in biochemical and blood count, including the leukogram data, which presented both a significantly decreased number of lymphocytes and an increased number of neutrophils + monocytes, evidencing the immune changes caused by the Ehrlich tumor.

## Conclusion

In conclusion, ZnPcS_4_-AN-mediated PDT enhanced the inhibition of tumor growth while producing practically no adverse effects and thus emerges as a very promising nanotechnology-based strategy for cancer treatment.

## Methods

### Chemicals

Doxorubicin, ketamin and xylazin were obtained as chlorhydrate. Lyophilized doxorubicin, sold as Doxofil 50 mg, was obtained from Ítaca Laboratórios Ltda (Rio de Janeiro, Brazil); ketamin, sold as Dopalen 100 mg/mL, was obtained from Ceva Animal Health Ltd (São Paulo, Brazil); and xylazin (Coopazine^®^ 20 mg/mL) came from Coopers (São Paulo, Brazil). Eosin methylene blue (Wright and Giemsa formulations) and trypan blue were purchased from Vetec Fine Chemistry Ltd (Rio de Janeiro, Brazil) and Sigma-Aldrich Co (São Paulo, Brazil), respectively. Zn(II) Phthalocyanine tetrasulfonate (Catalog # ZnPcS_4_-834) was obtained from Frontier Scientific Inc. (Utah, USA).

#### ***Albumin nanospheres containing zinc-phthalocyanine tetrasulfonate***

The new formulation of bovine serum albumin nanospheres loaded with phthalocyanine (ZnPcS_4_-AN) was based on previous work described by the São Paulo University PI# 0.803.473-7, with small changes. Briefly, the sunflower oil and the Span-70 were obtained from Aldrich Chemical Company, whereas bovine serum albumin was obtained from Calbiochem. The 2-propanol, analytical grade, was used as received. The albumin nanoparticle used in this study was prepared according to the method of heat denaturation using the mechanical stirring process at high-speed from an ultra-turrax setup to optimize the preparation of the nanoparticles. This method of preparation involves the formation of protein crosslink in suspension, with the initial formation of small drops of aqueous solution of albumin dissolved in an immiscible liquid phase (oily phase). The solidification of these drops through crosslinked covalent-bonding leads to the isolation of albumin nanoparticles. The entrapped material (in this case the ZnPcS_4_ as a photosensitizer) was initially dispersed in the albumin aqueous phase. The aqueous solutions of albumin (range from 50 to 300 mg/mL) and 300 μL of a stock solution 1.0 mM of ZnPcS_4_, were prepared in saline phosphate buffer (PBS) at pH = 7.4. At the same time 100 mL of the sunflower oil (containing 5% of Span 70, v/v) was initially added to a boiling flask of 100 mL. The ZnPcS_4_/albumin initial solution was dropped into the flask with continuous stirring using the ultra-turrax setup (5000 to 25000 rpm) for 20 minutes while the temperature of the system was fixed at 8°C. The mixture was then emulsified by ultrasound for some minutes at 250 W. In another boiling flask 100 mL of sunflower oil (containing 3% of Span 70) was pre-heated at 100°C under continuous agitation for 30 minutes. The initial emulsion obtained as described above was gently dropped directly onto the pre-heated sunflower oil in the boiling flask. After the formation of the initial emulsion the whole system was stirred at 10000 rpm for 10 minutes at 100°C. The final suspension was then cooled down to room temperature under continuous magnetic stirring. The obtained nanoparticles were washed with ethyl ether (3 × 30 mL) for oil separation, following centrifugation at 10000 rpm. After washing, the pellet containing the nanoparticles was lyophilized to remove any remaining water trace and stored at 4°C. The albumin-based nanosized structure was lyophilized and the material was stored and protected from light for a period of 3 months, without any morphological alteration. The prepared nanosized beads can be used for *in vitro* as well as for *in vivo* studies, using cell lines and animal models.

### Light source

A continuous low power (80 mW) diode laser (BWF light source - Tech in) operating at 670 nm, the wavelength of maximum optical absorption, adapted to an optical fiber, was used to excite the solution containing ZnPcS_4_-AN.

### Scanning electron microscopy

The external morphology of the bovine serum albumin nanospheres loaded with phthalocyanine, ZnPcS_4_-AN was examined by scanning electron microscopy after Au-coating using LEO-440 with tungsten filament. This technique is important to evaluate not only the particle size distribution, but also the morphology of the nanosized particles that will be used for *in vitro* studies.

### Fluorescence emission

The fluorescence analysis based on the emission spectra of ZnPcS_4_ and ZnPcS_4_-AN was performed using a Fluorolog 3 Spex from Jobin-Ivon (USA) with excitation fixed at 612 nm. The spectra were recorded in the range of 640 – 780 nm with excitation and emission slits fixed at 5/5 nm, respectively.

### Zeta potential and particle size

Sample ZnPcS_4_-AN was examined in regard to the Zeta potential and hydrodynamic diameter. The data were recorded as a function of time up to 30 days using the Malvern Zetasizer Nanoseries (Malvern Instruments, UK).

### Ehrlich tumor

In order to test the efficacy in tumor remission of ZnPcS_4_-AN mediated-PDT, combined or not with doxorubicin, ascitic-derived Ehrlich cells were used following the procedures previously reported [35-37]. The Ehrlich ascitic tumor, derived from a spontaneous murine mammary adenocarcinoma, was maintained in ascitic form by passages in Swiss mice by weekly intraperitoneal transplantation of 10^6^ tumor cells. The ascitic fluid was collected by intraperitoneal puncture using a sterile insulin syringe. Ascitic tumor cell counts were done in a Neubauer hemocytometer. The cells were found to be more than 99% viable by the trypan blue dye exclusion method.

#### ***Animals and experimental design***

All animal handling and procedures were carried out according to the international practices for animal use and care, and approved by the Animal Ethics Committee of the Institute of Biological Sciences, University of Brasilia, reference number 107748/2009. Female Swiss albino mice (11–12 weeks old) weighing 29 ± 1 g were obtained from the Multidisciplinary Center for Biological Research – CEMIB of the University of Campinas (Campinas-SP, Brazil). A total of 72 animals were acclimatized to laboratory conditions for two weeks before starting the study; mice were housed in plastic cages (6/cage) at room temperature (20 ± 2°C) in a 12 h light/dark cycle with lights on at 6 a.m. and free access to food and filtered water. After acclimatizing, animals were anesthetized by intraperitoneal administration of ketamine (80 mg/kg) and xylazine (10 mg/kg), both in the same syringe, in a final dose of 0.1 mL/30 g for tumor implantation. A volume of 20 μL (2.5 × 10^6^ viable cells) of Ehrlich ascitic tumor cell suspension was injected subcutaneously in the middle to lower posterior region of the ear (halfway from the bottom to the top of the ear, spreading to the line of contact with the head) for the solid form induction (a technical approach developed in our laboratory, unpublished data). Forty-eight hours after implantation of tumor cells, all mice had clinical tumor implanted on the ear. Animals were randomly divided into nine groups (8/cage), as detailed in Table 5: Negative control with no tumor implantation (NC group); Tumor inoculation and no treatment (CT group); Intratumoral injection of ZnPcS_4_-AN (ZnPC group); Tumor irradiated only with light from the laser setup (LS group); Intratumoral injection of ZnPcS_4_-AN followed by light activation (PDT group); Intratumoral injection of doxorubicin (Dox group); Intratumoral injection of ZnPcS_4_-AN and Dox (ZnPc/Dox group); Intratumoral injection of Dox followed by the same treatment as LS group (Dox/LS group); Intratumoral injection of ZnPcS_4_-AN and Dox followed by light activation as in PDT group (Dox/PDT).

**Table 5:** Description of experimental groups

**Group**	**Treatment**	**Treatment abbreviation**		**Experimental design**		
			**Tumor**	**ZnPcS**_**4**_**-AN**	**Laser**	**Doxorubicin**
1	Filtered water and no tumor implantation (negative control)	NC	**-**	**-**	**-**	**-**
2	Tumor inoculation and no treatment (control tumor)	CT	**+**	**-**	**-**	**-**
3	Intratumoral injection of 20 μL (0.5 mM) ZnPcS_4_-AN	ZnPc	**+**	**+**	**-**	**-**
4	Tumor irradiated only with light from a laser setup working at 670 nm, in a total dose of 100 J/cm^2^	LS	**+**	**-**	**+**	**-**
5	Intratumoral injection of 0.5 mM ZnPcS_4_-AN followed by light activation considering the same treatment as described for group 4	PDT	**+**	**+**	**+**	**-**
6	Intratumoral injection of 20 mg/m^2^ doxorubicin	Dox	**+**	**-**	**-**	**+**
7	Intratumoral injection of 0.5 mM ZnPcS_4_-AN and 20 mg/m^2^ Dox	ZnPc/Dox	**+**	**+**	**-**	**+**
8	Intratumoral injection of 20 mg/m^2^ Dox and the same treatment as group 4	Dox/LS	**+**	**-**	**+**	**+**
9	Intratumoral injection of 0.5 mM ZnPcS_4_-AN and 20 mg/m^2^ Dox and the same treatment as group 5	Dox/PDT	**+**	**+**	**+**	**+**

The energy fluence (100 J/cm^2^) used in this study was previously tested in a lingual tumor model [18]. To reach this energy density the tumor irradiation fields were standardized at 10 mm of circular diameter, the laser power was uniform and constant at 80 mW, and the time of laser irradiation was 16 minutes. The treatments were performed once a day every three days. During the experimental time, mice were clinically examined for clinical alterations.

### Procedures and measurements

Twenty-four hours after the last treatment, animals were anesthetized with a mixture of xilazin and ketamin according to the method described above. Blood samples (1 mL) collected by cardiac puncture were used to carry out hemogram and biochemical dosages of total bilirubin, direct bilirubin, indirect bilirubin, aspartate aminotransferase (AST), alanine aminotransferase (ALT), gamma glutamyl transferase (GGT), alkaline phosphatase, creatinine, and urea. Hemogram was processed in a multiple automated hematology analyzer for veterinary use, Sysmex pocH-100iV Diff (Curitiba, Brazil) calibrated for mice in microtubes containing EDTA as anticoagulant. Serum biochemical analyses were run on the automated chemistry analyzer ADVIA 2400 (Siemens), using the appropriate Advia chemistry reagents and protocols. Total bilirubin, bilirubin fractions and creatinine were measured by colorimetric assays; GGT by a colorimetric kinetic method; urea by an enzymatic colorimetric method; and AST, ALT and alkaline phosphatase by optimized kinetic methods. Euthanasia of the animals was carried out by cervical dislocation according to the AVMA Guidelines on Euthanasia [55].

Tumors, liver and kidneys were surgically removed. The width, length and thickness of tumors were measured using a digital pachymeter, and the volume was calculated according to Yanase et al. [56]. Afterwards, tumors and organs were fixed with 10% formalin for 24 h, transferred to 70% ethanol, embedded in paraffin using an automatic tissue processor (OMA^®^ DM-40, São Paulo, Brazil), cut to 5 μm of thickness in a Leica RM2235 manual microtome (Leica Microsystems, Nussloch, Germany) and stained with hematoxilin-eosin (HE) for histological analyses (light microscopy). A total of five histological sections with 100 μm distance between sections were analyzed per tumor sample. All histological sections were photographed with an MC 80 DX camera coupled to a Zeiss Axiophot light microscope, and tumor/necrotic areas were quantified using Image ProPlus 5.1 software.

### Statistical analysis

Statistical analysis was carried out using SPSS (Statistical Package for the Social Sciences) version 15.0. Data were expressed as mean ± SEM (standard error of mean) and values of p < 0.05 were considered statistically significant. The continuous variables were tested for normal distribution with Shapiro-Wilk. For animals’ body weight, the differences among groups were checked by ANOVA, while differences in other variables were investigated by ANOVA or Kruskal-Wallis test (when the data were not normally distributed). For significant ANOVA results, Bonferroni’s post-hoc test was chosen to carry out 2-to-2 comparisons between the treatments. For significant Kruskal-Wallis results, Mann–Whitney U test was performed to verify differences between the treatments (2-to-2 comparisons).

## Abbreviations

PDT: Photodynamic therapy; ZnPcS4-NA: Albumin nanospheres containing zinc-phthalocyanine tetrasulfonate; Dox: Doxorubicin; DDS: Drug delivery system; ROS: Reactive oxygen species; RBC: Red blood cells; HGB: Hemoglobin; HCT: Hematocrit; MCV: Mean corpuscular volume; MCH: Mean corpuscular hemoglobin; MCHC: Mean corpuscular hemoglobin concentration; RDW: Red cell distribution width; PLT: Platelet count; MPV: Mean platelet volume; P-LCR: Platelet large cell ratio; PDW: Platelet distribution width; AST: Aspartate aminotransferase; ALT: Alanine aminotransferase; GGT: Gamma glutamyl transferase.

## Competing interests

The authors declare that they have no competing interests.

## Authors’ contributions

FAP conducted all the biological tests; LLCE contributed to the biological tests and CEOC with tumor model development; MFAS and ALB contributed to the morphological and immunochemical analysis; OPM, ARS, ACT and PCM carried out the preparation and characterization of the sample; JPFL and RBA contributed to photodynamic therapy studies; ALMV was responsible for the statistical analysis; ZGML contributed to the work with the overall coordination of the project; FAP, ALMV, ACT and ZGML wrote the manuscript; and all authors contributed to the final revision of this manuscript.
